# Optical biosensing using particle diffusometry on thermoplastic microfluidic chips bonded using direct and indirect chip bonding methods

**DOI:** 10.1007/s10544-026-00810-4

**Published:** 2026-04-20

**Authors:** Julio A. Rivera-De Jesus, Alexander B. Memmer, Dong Hoon Lee, Tamara L. Kinzer-Ursem, Steven T. Wereley, Jacqueline C. Linnes, Melinda A. Lake-Speers

**Affiliations:** 1 Weldon School of Biomedical Engineering, Purdue University, West Lafayette, 47907 IN USA; 2 Department of Mechanical Engineering, Ohio University, Athens, 45701 OH USA; 3 Department of Mechanical and Aerospace Engineering, Case Western Reserve University, Cleveland, 44106 OH USA

**Keywords:** Point-of-care diagnostics, Materials testing, Rapid prototyping

## Abstract

Thermoplastics offer a scalable and cost-effective platform for fabricating microfluidic devices for point-of-care (POC) diagnostics. Among them, cyclic olefin polymer (COP) stands out due to its exceptional optical clarity, making it suitable for fluorescence-based biosensing. However, bonding COP layers without compromising mechanical strength or imaging quality remains a challenge. This study systematically evaluates four bonding techniques: thermal bonding, solvent bonding, UV-curable adhesives, and pressure-sensitive adhesives (PSA), using standardized 180-degree peel tests to quantify bond strength. PSA bonding exhibited the highest adhesion (0.2–0.8 N/mm), while thermal and solvent bonds were notably weaker (<0.05 N/mm). Plasma treatment improved bond strength and uniformity across most methods. We demonstrate the utility of these bonding techniques by fabricating multilayer microfluidic chips compatible with particle diffusometry (PD), an optical biosensing method that detects *Vibrio cholerae* DNA via changes in nanoparticle motion. Chips were assessed for both mechanical robustness and image intensity suitability for smartphone-based PD measurements. This work provides practical design criteria for selecting prototyping-compatible bonding strategies in the development of low-cost, optically clear microfluidic diagnostic platforms.

**Supplementary Information:**

The online version contains supplementary material available at 10.1007/s10544-026-00810-4.

## Introduction

Point-of-care (POC) microfluidic devices have become an essential tool in rapid diagnostics, offering decentralized testing with faster turnaround times compared to standard laboratory-based assays. Their ability to deliver results at or near the site of patient care has made them particularly valuable in resource-limited settings, field surveillance, and home-based diagnostics (Moehling et al. 2021; Yang et al. 2022). As the demand for portable, user-friendly diagnostic technologies increases, there is a growing need for materials and fabrication methods that can support scalable and cost-effective microfluidic device manufacturing. Thermoplastics have emerged as strong candidates for this purpose, given their affordability, mechanical stability, chemical resistance, and compatibility with high-throughput fabrication techniques such as roll-to-roll processing (Bartholomeusz et al. 2005) and injection molding (Kendall et al. 2014). These properties enable the development of disposable microfluidic devices suitable for mass production. Thermoplastics are not only favored for economic scalability but also for their compatibility with biosensing applications that require biocompatibility, minimal sample absorption, and resistance to heat and solvents. In contrast, polydimethylsiloxane (PDMS), while commonly used in academic settings for prototyping, faces multiple limitations for commercialization. These include the absorption or adsorption of small hydrophobic molecules (Clayton et al. 2019; Colbert et al. 2022) low barrier properties, and fabrication challenges due to the need for specialized molding setups and multi-step post-processing procedures (Colbert et al. 2021). Among thermoplastics, cyclic olefin polymer (COP) and cyclic olefin copolymer (COC) have gained prominence due to their excellent optical clarity, low autofluorescence, and chemical resistance (Tsao and DeVoe 2009). A key reason COP is widely used in optical applications is its high visible-range transparency, with reported light transmission >91% between 400–700 nm (Zeon Specialty Materials Inc 2026.). These features make them ideal candidates for optical biosensing platforms, particularly those relying on fluorescence-based detection (Tsao and DeVoe 2009). However, transitioning these materials into enclosed microfluidic devices introduces a major challenge: achieving reliable bonding between layers without compromising their mechanical integrity, channel geometry, or optical performance. Several bonding methods have been developed for thermoplastics and can be broadly categorized into direct and indirect bonding approaches. Direct bonding includes thermal bonding (Bhattacharyya and Klapperich 2007; Jena et al. 2011; Tsao et al. 2007; Colbert et al. 2022) where heat and pressure are applied to fuse two thermoplastic layers near or above their glass transition temperature, and solvent bonding (Wang et al. 2013; Keller et al. 2016; Wan et al. 2015; Wallow et al. 2007) which involves partial dissolution of the polymer surface with a solvent to create a chemical weld. While these approaches can produce seamless microchannels, they often risk deformation of delicate channel features or reduce optical clarity due to surface stress or solvent residues. Moreover, thermal bonding typically requires specialized presses or clamps and can be limited by variable bonding strength across different device designs. Indirect bonding techniques offer promising alternatives. These include ultraviolet (UV)-curable adhesives (Arayanarakool et al. 2010; Han et al. 2003; Satyanarayana et al. 2005; Hung et al. 2008) and pressure-sensitive adhesives (PSA) (Wang et al. 2013; Serra et al. 2017). UV-curable adhesives can be precisely deposited in defined areas and cured at room temperature, preserving channel features and material transparency (Giri and Tsao 2022). PSA provides a straightforward approach for device assembly and is easily patterned using rapid prototyping tools such as xurography and laser cutting. Although indirect methods may introduce additional interfaces that can interfere with optical sensing or create absorption layers, they have demonstrated successful application in proof-of-concept devices and are appealing for iterative prototyping. To further enhance bonding strength, plasma surface treatment is commonly employed to modify the thermoplastic surface. Plasma treatment can remove organic contaminants and temporarily alter the surface chemistry, increasing wettability and improving interfacial adhesion (Bhattacharyya and Klapperich 2007; Jena et al. 2011; Tsao et al. 2007). However, the benefits of plasma activation are time-sensitive and subject to hydrophobic recovery (Tsao and DeVoe 2009; Lee et al. 2005), limiting its effectiveness for devices requiring long-term storage or delayed assembly. In this study, we investigate the mechanical and optical compatibility of four bonding methods—thermal bonding, solvent bonding, UV-curable adhesive bonding, and PSA bonding—using cyclic olefin polymer substrates. Bond strength is quantified via 180-degree T-peel testing, while imaging performance is assessed through intensity measurements captured during particle diffusometry (PD) experiments. PD is a label-free optical biosensing method that measures the Brownian motion of nanoparticles to infer changes in fluid viscosity, typically induced by the presence of amplified nucleic acids (Moehling et al. 2020; Colbert et al. 2021; Clayton et al. 2019). We apply PD for the detection of *Vibrio cholerae* DNA using a smartphone-based platform described by (Moehling et al. 2020), which allows us to evaluate not only mechanical integrity but also optical performance in a functional diagnostic application. Through this comparison, we aim to provide practical guidelines for selecting suitable bonding strategies in the development of low-cost, robust, and optically transparent microfluidic chips for point-of-care diagnostics. Given the diverse constraints encountered in point-of-care deployment, our goal is not to prescribe a single optimal bonding method, but to provide quantitative comparisons of bond strength and optical performance across bonding strategies for COP microfluidic chips. These comparisons are intended to enable application specific selection based on available resources.

## Materials and methods

### Materials test strip and microfluidic chip design

To test the COP bonding strength, 188 $\mu$m thick COP (Zeonor, Tokyo, Japan), 75 mm x 10 mm (*L*X*W*) strips were bonded together in pairs. During the bonding preparation, one COP strip is left untreated by the adhesive method while the other is treated on half of its surface (37.5 mm) down the length (Fig. 3a). To test the biosensor compatibility, a U-shape chip was designed to hold an 11.3 or 15.0 $\mu$L fluid sample depending on if the Device Layer material was COP or PSA (Fig. 5). The U-shape chip comprises 3 layers: a Fluid Loading Layer which is a 188 $\mu$m COP part with 2 holes laser cut, a Device Layer made from 188 $\mu$m COP or 142$\mu$m PSA depending on the bonding method, and a blank 50 $\mu$m COP Imaging Layer through which the imaging is done. Once fully assembled, the microfluidic chip has a single inlet, a single outlet, and an imaging chamber to take the PD measurements.

### Xurography and laser cutting

The material testing strips are fabricated using xurography (Bartholomeusz et al. 2005), which uses a cutting blade to cut a pattern into a sheet of 188 $\mu$m COP (Zeonor, Tokyo, Japan) into 75mm x 10mm strips. We use a craft cutter, (Silhouette Cameo, Lindon, UT, USA). The U-shaped chips (Fig. 5) are also cut using xurography for the COP device layer or a laser cutter (VLS3.50, Universal Laser Systems Inc., Scottsdale, AZ, USA) for the PSA device layer at 90% power, 100% speed. Prior to bonding layers, the cut COP is cleaned with a 70% v/v ethanol solution and wiped dry with a Kimwipe.

### Plasma cleaning

For samples that have a plasma treatment applied, the chips are placed into a benchtop air plasma chamber (Harrick Plasma, Ithaca, NY, USA). The pressure is reduced using a vacuum pump (IDP-3, Agilent Technologies, Santa Clara, CA, USA), and the RF power is set to Medium or 10.5 W for 5 minutes. The samples are then removed holding the edges of the COP. Next the bonding method is applied to the plasma treated side of the COP before completing the bond with the untreated COP.

**Fig. 1:**
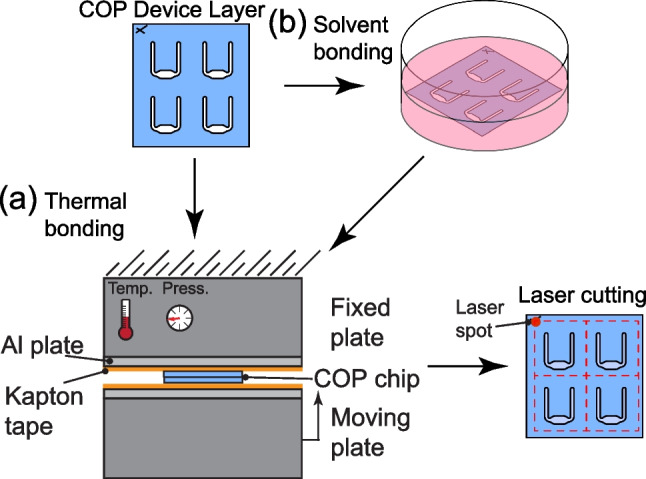
Direct bonding methods used in this paper. (**a**) Thermal bonding uses pressure and heat to complete the bonding process. (**b**) Solvent bonding has an additional step of etching the COP substrate in solvent mixture prior to applying heat and pressure such as in thermal bonding. For added bond strength, the bonded parts are diced into individual chips using a laser cutter, a method called laser fusion

**Fig. 2:**
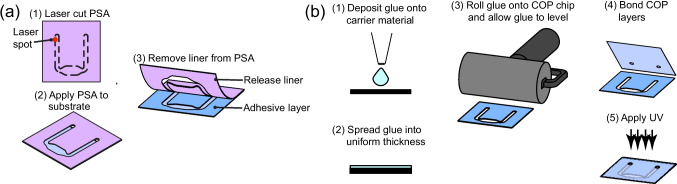
Indirect bonding methods used in this paper. (**a**) PSA bonding uses (1) a patterned layer of double-sided PSA, (2)-(3) that is bonded to the imaging layer, and the loading layer after removing the liners. (**b**) UV Glue bonding steps: (1) deposit UV curable glue onto substrate, (2) spread using a roller, (3) place the COP layers on the top and bottom and place under UV light for bonding

### Direct bonding methods

#### Thermal bonding

Thermal bonding is done using a Carver 4386 hydraulic press (Carver Inc, Wabash, IN, USA). The stacked COP layers are placed between 2 aluminum (1/8 inch thick) sheets with layers of Kapton tape (Fig. 1a) in the clamp. To bond the materials test strips, the clamp has a temperature of 120°C. A load of 1.2 tons is applied for 2 minutes, then the force is unloaded to rotate the aluminum sheet sample holders 180 degrees and reapply the load for 30 additional seconds.

For the biosensor testing, we used sheets with 4 U-shaped chips patterned on them to bond 4 microfluidic chips together at a time. The 50 $\mu$m layer was more sensitive to deformation at the temperature and pressure settings used for the materials strength testing. Therefore, we reduced the clamp temperature to 105°C and adjusted the loading weight to apply 1 ton for 30 seconds followed by 1.5 tons for 30 seconds, repeated thrice. Parts were removed and flipped 180 degrees between sets. We had two sets of chips, standard preparation where chips were bonded 1 chip at a time and laser fused chips. The laser fused chips had additional processing done in that we bonded 4 chips at a time and then we diced the bonded layers into individual chips using the laser cutter at 95% power, 100% speed through 2 laser passes.

#### Solvent bonding

**Fig. 3:**
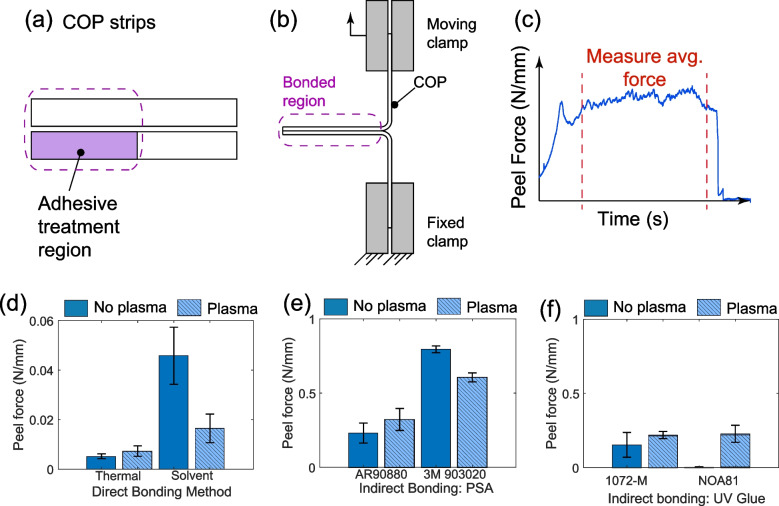
Mechanical characterization using T-peel test to separate 2 bonded COP strips. (**a**) COP strips bonded together where 1 strip is treated directly with adhesive while the other is not. The treated region is a 375 mm$^2$ rectangle. (**b**) Mechanical tester schematic. (**c**) Representative plot of peel force over time as the 2 strips are pulled apart by the mechanical tester. Plot of average peel force measurements from the tensile test machine for $n=3$ for (**d**) direct bonding methods thermal and solvent, (**e**) indirect bonding with PSA, and (**f**) indirect bonding with UV-glue

Solvent bonding (Fig. 2b) is performed using decahydronaphthalene (decalin) diluted to 35% v/v in ethanol following similar methods as (Kendall et al. 2014). The COP part for materials testing (Fig. 3a) is treated with solvent by dipping one of the COP strip up to a halfway mark into the solvent mixture for 2 minutes. Next, the sample is sprayed off with ethanol, dried with N$_2$, and then loaded onto the heat press that applies load of 1.2 tons and is preset to 120°C. Although solvent bonding can allow for lower temperature bonding, the bond strength is weak. The layers for the U-shaped chip for biosensor testing (Fig. 5) are all cleaned using ethanol and N$_2$ dry but only the device layer (Fig. 5a) is treated with the solvent mixture for 2 minutes to avoid clouding the imaging layer. Here, similar to in thermal bonding, we used standard preparation where chips were bonded 1 chip at a time or with a laser fusion method to dice a sample that has 4 U-shapes patterned into individual chips after bonding. A stir bar is used at 200 rpm to maintain uniformity of the mixture during treatment. Similar to the thermal bonding for the biosensing chip, the 50 $\mu$m COP is sensitive to pressure and temperature so the same method is applied to modify temperature and pressure to match the biosensor thermal bonding process.

### Indirect bonding methods

#### Pressure sensitive adhesive (PSA) bonding

Two types of PSAs are evaluated: Adhesives Research (AR) 90880 (Adhesives Research, Glen Rock, PA, USA) and 3M 903020LE (3M, St. Paul, MN, USA). The PSA is through-cut using a laser cutter. After cutting, one of two of the release liner layers are removed and the PSA is applied to the COP substrate. A uniform bond is created by rubbing the PSA using tweezers. Then the top release liner is removed and another COP layer placed on top. After removal of the release liners, the AR90880 has a nominal thickness of 142 $\mu$m and the 3M 903020LE has a nominal thickness of 210 $\mu$m. The materials testing COP (Fig. 3a) had a piece of adhesive sandwiched between 2 pieces of COP to complete the bond, while the biosensor chip (Fig. 5) had the device layer cutout directly from the PSA to bond the Fluid Loading and Imaging layers together (Fig. 5) PSA biosensor chips were constructed only using AR 90880.

#### Ultraviolet curing

Two ultraviolet (UV)-curable adhesives–Norland Optical Adhesive (NOA) 81 (Norland Products, Jamesburg, NJ, USA) and Dymax 1072-M (Dymax, Torrington, CT, USA)—were evaluated for bonding performance. We also examined the effects of plasma cleaning the substrates prior to adhesive application

For material test strips, UV adhesive was applied by first drawing 150µL into a micropipette and depositing it onto a carrier sheet of aluminum foil. A roller was used to spread the adhesive approximately 3 cm outward from the center in four back-and-forth passes. A clean COP strip was then partially adhered to the spread adhesive, with a second COP strip placed flush against it to complete the sandwich. Prior to UV curing, samples were left at room temperature (25$^\circ$C) for 24 hours to allow leveling and adhesive wetting. After curing the chips were baked for 24 hours at 50$^\circ$C to increase bond strength.

UV curing was performed using a CL-3000L UVP crosslinker (Analytik Jena, Jena, Germany) with 365nm light for 1 minute. The biosensor chips were fabricated one at a time using a “stamp-and-stick” method. The device layer (Fig. 5a) was coated with 150µL of NOA81 adhesive across a 30$\times$25mm area, which was then flattened with a roller. To prevent damage to the functional areas of the biosensor chip during stamping, thermoplastic handles were added to the xurography cut file. This design allowed for consistent adhesive transfer prior to final bonding of the imaging or fluid-loading layers.

### T-Peel test

Bond strength of the materials testing strips is evaluated using a Test Resources 100Q material testing machine to perform a 180°Peel, or T-Peel, test. The unbonded portions of materials testing strips are bent to form a T-shaped figure (Fig. 3b). After securing the chip within the grips, the materials testing machine pulls the two COP strips apart from one another at an applied tensile load of 50 N/min with a data log rate of 64 Hz. For evaluation, the raw data collected from each chip trial is exported and analyzed to find a steady-state average of the peel force (Fig. 3c).

### Biocompatibility and imaging compatibility test microfluidic chip preparation

To reduce the possibility of non-specific amplification from environmental sources all chips used for amplification testing are exposed to UV light (Class II Biosafety Cabinet, Labconco Corporation, Kansas City, MO) for five minutes to sterilize the fluidic channel prior to loading any biological samples. PSA chips have an extra degas step for fifteen minutes in a benchtop vacuum chamber to form a tighter seal between the PSA and COP layers and minimize air bubbles. To properly load the sample and rapidly achieve ideal fluid distribution through the channel; the reaction mixture is placed on the channel inlet and aspirated through the outlet utilizing a manual PDMS push button (Im et al. 2016). The chips are sealed by placing a 0.5x0.5 cm piece of a sealant tape for optical assays (Bio-Rad, Hercules, CA, USA) on both the inlet and outlet of the microfluidic device. The chips are then heated on a hot block at 64°C for 30-45 minutes to run the LAMP reaction. A range of time was evaluated to avoid amplification inhibition, while Moehling et al. consistently amplified for *V. cholerae* in less than 30 min (Moehling et al. 2020). Finally, to extract the sample after on-chip amplification, the sealant is carefully removed from the inlet and outlet ports, with a pair of tweezers and a PDMS button (Im et al. 2016) (see Fig. S4) is used as manual pump to push the fluid out of the chip. The fluid is collected in a pipette tip and loaded into a PCR tube before loading into the gel electrophoresis set up. Amplicon validation served as the last line of control for our samples. Given the possibilities of false negatives and false positive throughout extensive sample handling, the extracted sample was verified to validate our results prior to discarding it. With this in mind, any samples that displayed a band on agarose gel electrophoresis were classified as true positives, even if particle diffusometry results were within the range typically associated with negative screening.

### Bacteria culture

We culture bacteria following the methods described in (Moehling et al. 2020). We used toxigenic *V. cholerae* strain N16961 (O1 serogroup) stored at -80°C. A small sample of frozen cholera is extracted using a pipette tip and loaded into 3 mL of Lysogeny Broth (LB) culture medium and cultured for 24 hours at 37°C in an orbital shaker at 300 rpm (Thermo Fisher, Waltham, MA). During sample preparation, the culture is diluted to an OD600 of 1 during sample preparation, corresponding to 5$\times$10$^8$ bacteria cells/mL.

### Loop-mediated isothermal amplification (LAMP)

LAMP reactions are prepared according to the methods described in (Moehling et al. 2020). including LAMP primers. These primers target cholera toxin A (ctxA) gene of toxigenic *V. cholerae* strains. The reactions use pond water as a sample matrix, collected from a local lake. The negative controls (NTC) were spiked with 2 $\mu$L of template or water (molecular biology water, Invitrogen, Carlsbad, CA) for the negative control (NTC). In this work, amplification is performed on the chip at 64°C for bonded COP devices, placing additional demands on sealing and optical performance relative to imaging-only chip formats. To validate amplification on the device, after imaging the chips, the fluid is extracted as mentioned in section 2.7 and placed on a 1.5% EtBr agarose gel for analyzing. 10 $\mu$L of the fluid was extracted and combined with 2 $\mu$L of 6X purple loading dye (NEB, Ipswich, MA) along with 10 $\mu$L Fast DNA Ladder (NEB, Ipswich, MA) and run at 100V for 1 hour. The EtBr gels are imaged using an ultraviolet light gel imaging system (c400, Azure Biosystems, Dublin, CA) with 15-second exposure and 302UV setting. The master mix is also validated through LAMP in an Applied Biosystems 7500 Real-Time PCR System (Foster City, CA).

### Particle diffusometry

Particle diffusometry (PD) measures the Brownian motion of nanoparticles in solution as a measure of the change in fluid viscosity that is increased by the polymerization of pathogen DNA during nucleic acid amplification (Clayton et al. 2019). Series of images extracted from a smartphone video are auto- and cross-correlated to measure pixel displacement as done in prior work (Moehling et al. 2020; Colbert et al. 2021). Briefly, PD measures Brownian motion of particles in a solution volume and calculates the experimental diffusion coefficient, *D*:

1 $$\begin{aligned} D=\frac{s_c^2-s_a^2}{16M^2 \Delta t} \end{aligned}$$

where $s_c$ is the cross-correlation value, $s_a$ is the autocorrelation value, *M* is the magnification, and $\Delta t$ is the time step between images (Moehling et al. 2020; Clayton et al. 2019; Colbert et al. 2021). *D* is related to the fluid viscosity, $\eta$, by the Stokes-Einstein equation:

2 $$\begin{aligned} D=\frac{kT}{6\pi {\eta }a}, \end{aligned}$$

where *k* is the Boltzmann constant, *T* is the absolute temperature in Kelvin, and *a* is the hydrodynamic radius (Moehling et al. 2020; Clayton et al. 2019; Colbert et al. 2021).

In calculations, the Boltzmann constant was taken as $k = 1.38\times 10^{-23}$ J/K. The absolute temperature was set to $T = \text {298}$ K. The particle hydrodynamic radius was $a = \text {200}$ nm . For PD analysis, videos were recorded at 30 fps and reduced to an effective 15 fps by extracting every other frame, resulting in $\Delta t = 0.067$ s.

### Smartphone imaging and video recording

The smartphone platform used is fully described (Moehling et al. 2020). Briefly, the platform comprises a ball lens positioned under the iPhone 6 camera lens in a mount housed in a 3D printed part. A blue laser diode (Laserland, Wuhan, China) excites the green fluorescent beads (400 nm streptavidin coated Dragon Green fluorescent particles, Ex 480/Em 520 nm, Bangs Laboratories, Inc. Fishers, IN). Videos are recorded for 23 seconds and analyzed using a custom built app to measure the diffusivity (Eq. 1). The PD measurements may be sensitive to differences in image intensity. Image intensity was quantified using ImageJ to determine if the videos recorded for PD were acceptable for accurate screening. Using the rectangle drawing tool, we defined a region of interest (ROI) within screenshots of the sampled recordings, and the intensity was measured as mean gray value. Some variation in intensity is due to the adjustment of the image intensity on the iPhone before recording; however, the dim videos could not be sufficiently increased in brightness for accurate PD analysis. Based on the measurements and a qualitative analysis, an acceptable threshold between 13 and 55 was set for sufficient quality for PD measurements. This acceptance window was selected empirically to ensure adequate signal-to-noise and contrast for reliable auto-/cross-correlation, while avoiding saturation or insufficient particle visibility that degrades PD displacement estimates. Videos outside the acceptable intensity range were excluded. Due to this, resulting sample sizes were reduced and not uniform across bonding groups, limiting statistical power for PD separation in this dataset.

### Statistical analysis

To statistically determine if there is any difference in both positive and NTC groups, a one-way ANOVA post hoc Dunnett’s test was performed comparing the coefficient of diffusion values with a 95% confidence interval. With this method, there were no statistically significant differences between the diffusion coefficient for both groups (p-value = 0.11). Consequently, there is strong evidence supporting the application of this assay will not yield significant results to rule in/out the presence of the pathogen in the sample. However, it is important to note that due to used exclusion criteria sample sizes are not consistent. Further studies should be done with increased sample size to address the viability of the assay.

## Results and discussion

### Peel testing results

We first evaluated the mechanical integrity of each bonding method using 180-degree T-peel tests. The peel force was used as a quantitative measure of bond strength between COP layers. Among all methods tested, PSA bonding (particularly 3M 903020LE) demonstrated the highest peel strength, with values ranging from 0.2 to 0.8 N/mm. This aligns with PSA’s known capacity to form strong adhesive interfaces via viscoelastic contact and energy dissipation, which contributes to its durability under mechanical stress.

In contrast, thermal and solvent bonding methods resulted in the weakest adhesion (Fig. 3d-f), both falling below 0.05 N/mm. These values are consistent with prior reports of limited interfacial interaction and potential deformation at bonding temperatures near the glass transition point (Bhattacharyya and Klapperich 2007). Plasma treatment improved bond strength for thermal and UV-glue methods, confirming previous findings that surface activation enhances adhesion (Jena et al. 2011; Tsao et al. 2007). However, it had a mixed effect on PSA bonding, slightly reducing strength in some cases, likely due to surface over-oxidation.

### Image intensity results

**Fig. 4:**
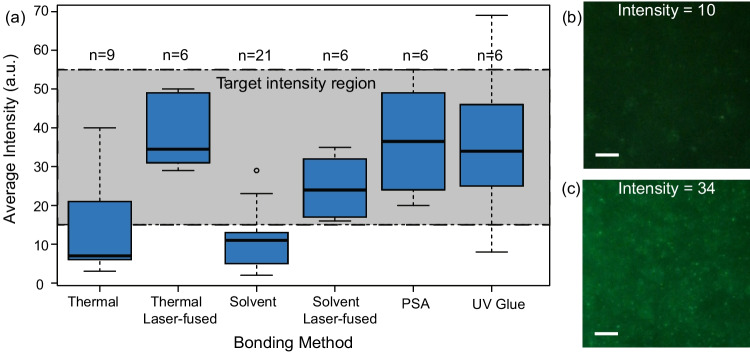
(**a**) Average intensity measured using ImageJ, separated by bonding method. The target intensity region indicates between what range the images were not too bright or too dark for diffusometry measurements. Examples of images from solvent bonded chips to show improvement in image intensity with laser fusing. (**b**) Intensity measured as 10 outside of acceptable threshold and (**c**) Intensity measured as 34 within acceptable threshold. Scale bar shows 100 $\mu$m

To determine the suitability of each bonding method for PD, we analyzed the brightness of images captured from PD videos using ImageJ (Fig. 4). Clear visualization of nanoparticle motion is essential for reliable PD, and image brightness within a target range was considered optimal.

PSA and UV-glue bonded chips consistently yielded videos within the optimal intensity range with minimun outliers, supporting their compatibility with optical biosensing. Solvent and thermal bonded chips showed greater variability and more frequent exclusion due to under- or overexposure. Notably, laser fusion significantly improved the brightness consistency in thermal and solvent bonded chips, likely due to increased seal uniformity and consequently reduced evaporation. Figure S3 shows a representative heating test comparing thermally bonded chips with and without laser fusion. Although this test was not performed across all bonding conditions, it qualitatively demonstrates improved sample retention in chips that underwent laser fusion compared to untreated controls.

### Particle diffusometry

Positive samples showed a general trend toward lower diffusion coefficients compared to negative controls; however, this difference did not achieve statistical significance, likely due to small and inconsistent sample sizes, especially within the thermal and solvent bonding groups (Figure S1). Despite these limitations, optical clarity was maintained across all bonding methods. However, bond strength inconsistencies in several chips led to minor sample loss, which may have contributed to variability in PD results.

As part of the validation process, all chips underwent post-imaging gel electrophoresis to confirm the presence of nucleic acid amplicons. Although particle diffusometry (PD) was used as a label-free, real-time screening method to detect changes in fluid viscosity caused by amplification, gel electrophoresis served as a definitive confirmation of the presence of the target DNA. Accordingly, for the purposes of this study, samples were considered truly positive only if they exhibited banding patterns on agarose gel, regardless of PD output.

A subset of samples was also amplified using a real-time PCR system for assay verification (Fig. 6). Although amplification was observed in some cases, variability in banding patterns limited direct correlation with PD values. Prior work has established PD performance under controlled imaging conditions and a standard platform. Clayton et al. (2019, ) provided extensive characterization of the PD assay with a microscope using a glass-PSA chip for imaging only, thus assay results were independent of the imaging chip. Moehling et al. (2020) demonstrated translation of PD from microscope imaging to a smartphone platform with strong diagnostic performance in a large double-blinded study. Colbert et al. (2021) extended the PD platform for malaria detection in blood samples; however, again the chips were used for imaging only. By contrast, Colbert et al. (2022) reported on-chip amplification in U-shaped thermoplastic chips and noted that achieving an airtight seal was challenging for thermal bonding, emphasizing the importance of this step for reliable diffusion-based measurements. Building on this body of work, the present study specifically evaluates COP bonding methods as the experimental variable in an imaging-sensitive workflow; therefore, bonding dependent factors such as seal integrity, and interface uniformity can contribute to increased variability and reduced statistical separation under limited sample sizes.

Table 1 summarizes the comparative performance of all bonding methods evaluated in this study. Taken together, these findings suggest that solvent bonding combined with laser fusion offers the most balanced approach in terms of optical compatibility and PD performance, while UV adhesive bonding yields high clarity but variable mechanical strength.

**Fig. 5:**
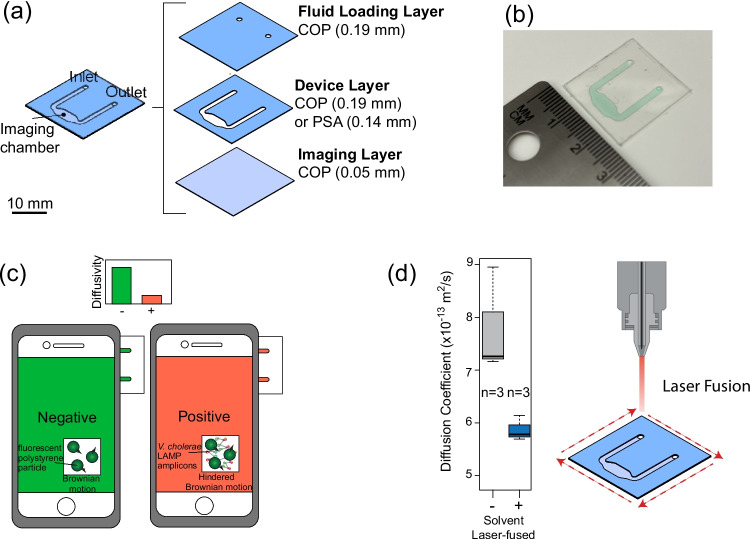
(**a**) U-shape channel assembly used for biosensor testing. (**b**) Photograph of the chip filled with food coloring. (**c**) The microfluidic chip is inserted into the side of the smartphone case to take video of the imaging chamber in the chip and tell a positive or negative result. (**d**) Particle diffusometry results for bonded U-shape chips. Results include samples for laser-fused solvent bonded chips

Notably, chips fabricated using solvent bonding combined with laser fusion consistently achieved acceptable imaging intensity and supported accurate screening, suggesting this bonding strategy may offer a promising balance between mechanical integrity and optical performance in PD-based diagnostic applications (Fig. 5d).

**Fig. 6:**
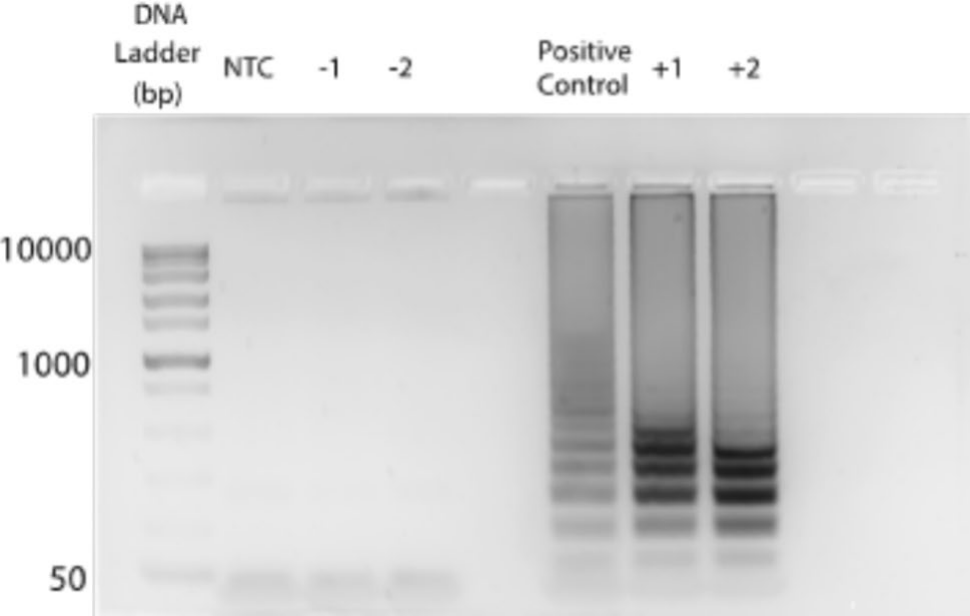
Agarose gel displaying banding pattern indicative of LAMP amplification of 10$^8$ cell/mL

**Table 1:** Comparison of COP bonding methods across key performance categories

Category	Thermal	Solvent	UV Glue	PSA
Peel Strength	< 0.05 N/mm	< 0.05 N/mm	0.2–0.6 N/mm	0.2–0.8 N/mm
Optical Clarity	Often too dim	Clouding possible	Within range	High, consistent
Plasma Effect	Improved	Improved	Improved	Mixed
Ease of Use	Moderate	Moderate	Moderate	High
Laser Fusion	Recommended	Recommended	Not needed	Not needed
PD Suitability	Limited (seal)	Limited (leakage)	Good	Excellent

Selection of an optimal bonding method is application-dependent. For rapid assembly and consistent fluorescence imaging, PSA and UV-curable adhesive approaches performed most reliably; when minimizing consumables and using widely accessible tooling, thermal or solvent bonding may be preferred, with laser fusion serving as a practical post-processing step to improve sealing and optical consistency. Therefore, Table 1 is intended as a selection framework rather than a prescriptive ranking.

## Conclusions

This study evaluated four bonding methods thermal, solvent, UV-curable adhesive, and PSA for compatibility with optical biosensing in COP microfluidic chips. Our results showed that PSA bonding produced the highest adhesion at 0.2–0.8 N/mm, followed by UV-curable adhesive bonding at 0.2–0.6 N/mm, whereas thermal and solvent bonding were consistently lower<0.05 N/mm (Table 1). For optical suitability in particle diffusometry (PD), videos were considered acceptable when the mean gray intensity fell within 13–55 as shown in Fig. 4; PSA and UV-glue chips most consistently met this threshold, while thermal and solvent methods showed greater variability, with laser fusion improving brightness consistency in both direct-bonded approaches. Laser fusion was used here as a pragmatic post-processing step to improve sealing and imaging consistency using the laser settings specified in Section 2.4.1. In-depth optimization of laser parameters and their effects on COP material response will be investigated in future work.

In PD testing, positive samples trended toward lower diffusion coefficients than negative controls, but the difference was not statistically significant (one-way ANOVA, p = 0.11) under the sample sizes evaluated. Overall, PSA and UV-glue provide the strongest bonds and most consistent optical performance, while solvent bonding combined with laser fusion offered the best balance of practical robustness and imaging suitability for rapid prototyping. These results provide quantitative guidance for selecting COP bonding strategies where both mechanical stability and optical readout quality are required, and future work will focus on improving repeatability and assessing long-term stability and scalability.

## Supplementary Information

Below is the link to the electronic supplementary material.

Supplementary file 1 (pdf 24232 KB)

## Data Availability

Data sets generated during the study are available from the corresponding author on reasonable request.
