# Supplementary material for: Optical biosensing using particle diffusometry on thermoplastic microfluidic chips bonded using direct and indirect chip bonding methods
Source: Biomed Microdevices. 2026 Apr 20;28(2):29. doi: 10.1007/s10544-026-00810-4 (PMC13095912; doi:10.1007/s10544-026-00810-4)
Supplement: Supplementary file 1 — (pdf 24232 KB) [file 10544_2026_810_MOESM1_ESM.pdf]

Optical biosensing using particle diffusometry on thermoplastic microfluidic chips bonded using direct and indirect chip bonding methods

Julio A. Rivera-De Jesus[1], Alexander B. Memmer[1], Dong H. Lee[1], Tamara L. Kinzer-Ursem[1], Steven T. Wereley[2], Jacqueline C. Linnes[1], Melinda A. Lake-Speers[3*]

[1]Weldon School of Biomedical Engineering, Purdue University, West Lafayette, 47907, IN, USA.
[2]Department of Mechanical Engineering, Ohio University, Athens, 45701, OH, USA.
[3]Department of Mechanical and Aerospace Engineering, Case Western Reserve University, Cleveland, 44106, OH, USA.

*Corresponding author(s). E-mail(s): mal259@case.edu;

A Particle Diffusometry

Due to fabrication variability and availability of confirmed positive samples, sample sizes varied across bonding conditions. Although this limits direct statistical comparisons, observed trends remain informative for evaluating performance consistency. To evaluate the consistency and accuracy of particle diffusometry (PD) measurements across bonding methods, we compared diffusion coefficients from samples stratified by PD outcome (positive or negative) and validated against gel electrophoresis results (Figure S1). Across bonding methods, diffusion coefficients varied widely, with some overlap between PD-positive and PD-negative classifications. Notably, chips bonded with solvent alone or with UV adhesive tended to show tighter distributions and clearer separation between positive and negative PD outcomes, supporting improved imaging and flow stability. In contrast, thermal bonding exhibited more variability, possibly due to inconsistent bonding strength or optical distortion from thermal stress. These results emphasize the importance of fabrication method in maintaining assay reproducibility and highlight the need to correlate PD data with confirmatory molecular assays.

B Challenges

During the evaluation of the biosensor chips, a recurring challenge was the presence of air bubbles within the fluidic channels and between plastic layers, which compromised the reliability of particle diffusometry measurements. These air pockets, visible in Figure S2, may have originated from multiple sources, including sample loading, heating, chip manipulation, or incomplete lamination during bonding. Two distinct types of bubbles were observed: (1) fluid-phase air bubbles (represented in enclosed red regions), likely introduced during loading, handling, or heating and (2) interfacial air pockets (represented in enclosed blue regions in Figure S2A-C), suggestive of delamination or bonding defects. Both bubble types could potentially lead to critical artifacts from allowing bubbles to form in the main fluid channel. Notably, chips with minimal visible interference in the imaging chamber (Figure S2C) exhibited more uniform particle motion and higher-quality imaging, although subtle regions of delamination were still present away from the fluid channel. Therefore, minimizing air entrapment—through, but most importantly establishing a strong bond is crucial to ensure consistent assay performance.

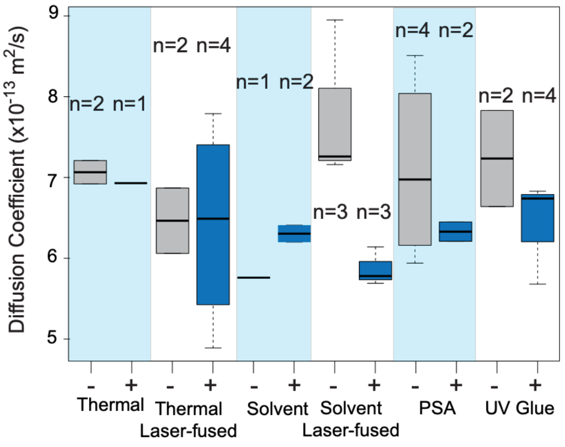

Fig. S1 Diffusion coefficient measurements across bonding methods and PD outcomes. Boxplots represent results for solvent bonding, thermal bonding, laser + solvent bonding, UV adhesive bonding. Blue bars indicate samples confirmed as positive by gel electrophoresis; gray bars indicate negatives. (PD) outcome (-: negative by PD, +: positive by PD), with sample sizes indicated. Variability in PD measurements across bonding methods was observed, with greater consistency in solvent and UV adhesive bonded chips. Misalignment between PD and gel results highlights the influence of bonding quality and sample handling on PD performance.

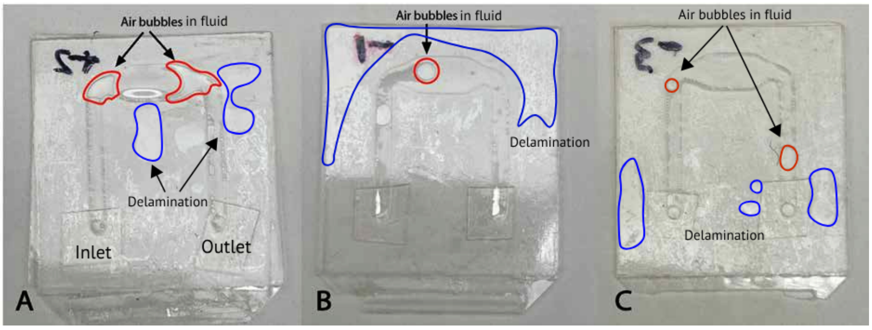

Fig. S2 Solvent bonded chips (A) Here we can observe the distinct artifact that form on the chip post amplification and affect image processing. In (B) we can see a bubble formed in the imaging chamber, this induces flow in the channel. This flow affects the numerical calculation of the diffusion coefficient with the addition that here we are limited to the quality in observed in the restricted region due to the air bubbles. These and combinations of these scenarios affect particle diffusometry measurements. (C) Loaded chip with minimal artifacts in the ROI, still affected from air bubbles and delamination in other parts of the chip. Note: figure brightness and contrast were adjusted to better highlight air bubble features; light regions in the bonded plastic represent delamination.

A main point already mentioned was the further delamination that could occur through heating poorly bonded chips which would lead to undesired evaporation. In the main article we addressed this issue and propose to laser fused bonded chips to mitigate sample evaporation. In Figure S3 we can qualitatively observe in the improvement in sample retention after heating of laser-fused thermal bonded chips.

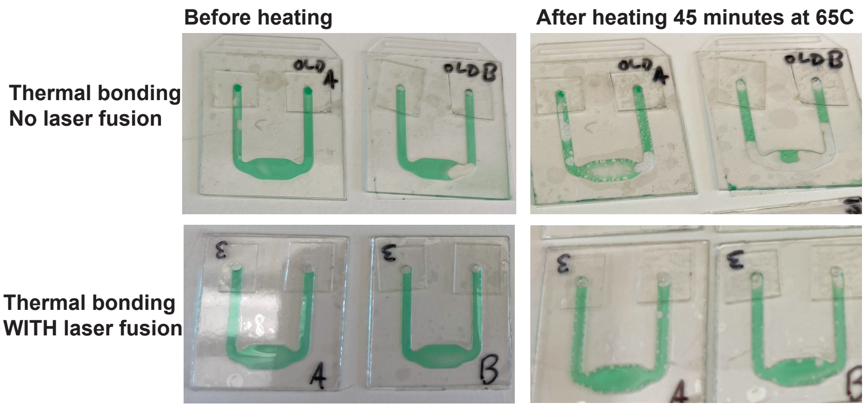

Fig. S3 Laser-fused Thermal bonded chips loaded with green food coloring before and after heating

C PDMS push button

To enable manual fluidic loading of the biosensor chip, a PDMS-based push button mechanism was fabricated (Figure S4). This feature was developed to allow consistent sample injection or ejection, before sealing, without requiring external pressure systems. The push button consisted of a soft elastomeric cylindrical cap aligned at the fluid outlet and actuated through finger-applied pressure.

PDMS components were fabricated using a standard soft lithography process. Briefly, a 10:1 base-to-curing agent mixture of Sylgard 184 (Dow Corning) was thoroughly mixed and degassed under vacuum for 15-30 minutes. The mixture was then poured over a laser-cut acrylic master or mold defining the push button shape and cured at 65°C for 2 hours. After curing, the PDMS layer was peeled from the mold and individual buttons were cut using a razor blade. These sub assemblies were then adhered to the inlet or outlet of the biosensor chip, forming a mechanically actuated interface for fluid control.

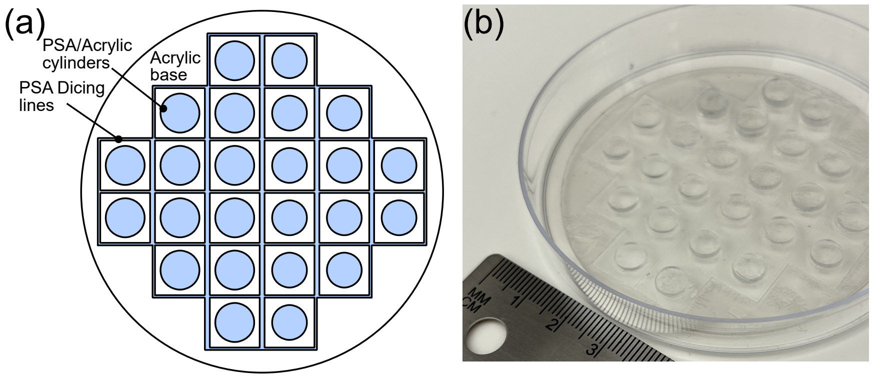

Fig. S4 For fluidic loading, a push button fabricated of PDMS was used.(A) Sketch of PDMS push button. (B) PDMS casted on the acrylic mold button mold.
